# Bridging gaps and seeding futures: A synthesis of soil salinization and the role of plant-soil interactions under climate change

**DOI:** 10.1016/j.isci.2024.110804

**Published:** 2024-08-23

**Authors:** Hao Tang, Lei Du, Chengcheng Xia, Jian Luo

**Affiliations:** 1 Key Laboratory of Land Resources Evaluation and Monitoring in Southwest, Ministry of Education, Sichuan Normal University, Chengdu 610068, China; 2 School of Geography and Tourism, Chongqing Normal University, Chongqing 401331, China; 3 Inner Mongolia Key Laboratory of River and Lake Ecology, School of Ecology and Environment, Inner Mongolia University, Hohhot 010021, China

**Keywords:** Soil science, Agricultural science, Agricultural soil science

## Abstract

Soil salinization, exacerbated by climate change, poses significant threats to agricultural productivity, land restoration, and ecosystem resilience. This study reviews current knowledge on plant-soil interactions as a strategy to mitigate soil salinization induced by climate change, focusing on their role in soil salinity dynamics and tolerance mechanisms. The review examines how alterations in hydrological and temperature regimes impact soil salinity and how plant-soil mechanisms—such as salt exclusion, compartmentalization, and plant-microbe interactions—contribute to salinity mitigation. This, in turn, enhances soil quality, fertility, microbial diversity, and ecosystem services. The analysis identifies a growing body of research and highlights key themes and emerging trends, including drought, microbial communities, and salt tolerance strategies. This study underscores the critical role of plant-soil interactions in sustainable salinity management and identifies knowledge gaps and future research priorities, advocating for plant-soil interactions as a crucial pathway for improving ecosystem resilience to salinity stress amid climate change.

## Graphical abstract

**Figure:**
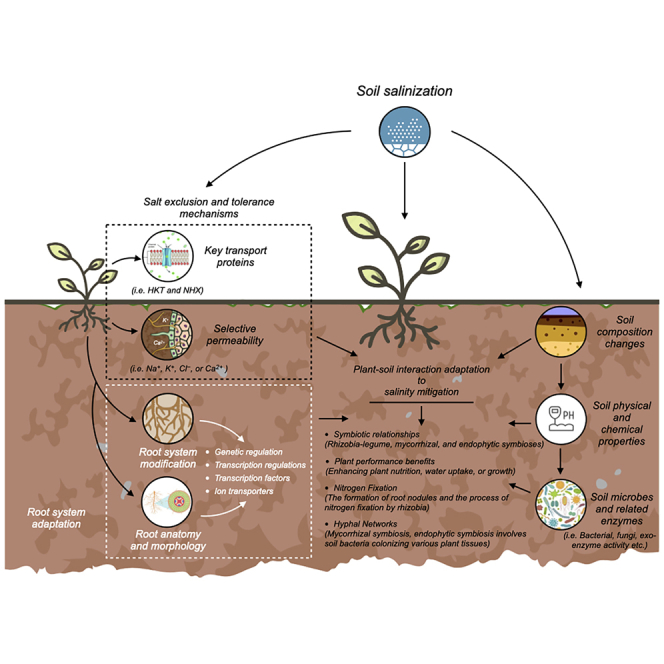

## Introduction

Soil salinization is a global problem that affects more than 800 million hectares of land, reducing agricultural productivity and threatening food security.^1^^,^^2^ It is caused by the accumulation of soluble salts in the soil, which can adversely affect soil physical, chemical, and biological properties, as well as plant growth and development.^3^^,^^4^ Soil salinization can be caused by natural or anthropogenic factors, such as arid and semi-arid climates, irrigation with saline water, poor drainage, fertilizer application, or land use changes.^5^^,^^6^ The interplay between evaporation, precipitation, irrigation practices, and the quality of irrigation water plays a pivotal role in the salinization process, making certain regions, especially arid and semi-arid areas, particularly vulnerable.

The impacts of climate change exacerbate the challenges posed by soil salinization. With the global climate system undergoing rapid changes, including rising temperatures, shifting precipitation patterns, and increasing frequency of extreme weather events, the dynamics of soil salinization are also being altered.^5^^,^^7^^,^^8^ These climatic shifts contribute to the acceleration of soil degradation processes, including salinization, thereby amplifying the stress on agricultural systems and natural ecosystems.^2^^,^^9^^,^^10^ The repercussions of soil salinization under climate change are far-reaching, impacting food security, livelihoods, and the health of terrestrial ecosystems.^11^

**Figure 1:**
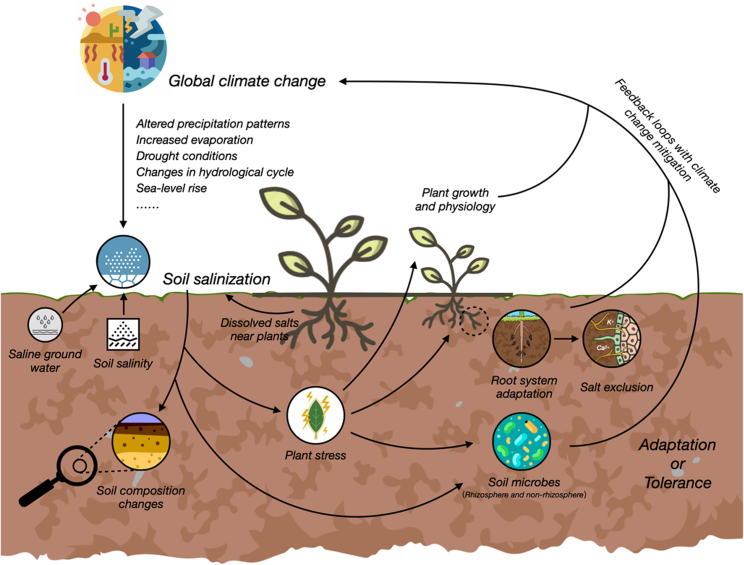
Soil Salinization Processes and Impacts This figure outlines the processes leading to soil salinization, encompassing causes, the influence of global climate change, effects on soil composition, plant stress, root adaptations, and the role of soil microbes. It also highlights the interconnected feedback loops within this environmental challenge.

In this context, understanding the interactions between plants and soils emerges as a crucial aspect of mitigating the adverse effects of soil salinization.^2^ Plant-soil interaction is the dynamic interplay between plants and soil biota, mediated by various factors such as root exudates, soil organic matter, nutrients, and water.^12^ This interaction plays a crucial role in mitigating soil salinization and enhancing soil health by influencing salt dynamics, water relations, nutrient cycling, microbial activity, and ecosystem services.^1^^,^^13^ Strategies for plant-soil interaction include salt exclusion and tolerance mechanisms in plants, root system adaptations for salt dilution and excretion, and plant-microbe interactions for salinity management.^14^ These strategies have been applied or proposed for sustainable soil salinity management in various contexts such as agriculture, land restoration, or ecosystem conservation.^2^^,^^8^^,^^10^^,^^15^^,^^16^ They offer multiple benefits for soil salinization mitigation, such as reducing salt stress, improving plant performance and quality, restoring soil functions and services, and developing climate-resilient ecosystems (Figure 1).

Given the growing body of literature on soil salinization and plant-soil interactions, there is a critical need to synthesize, analyze, and understand the current research landscape. The objectives of our review study are 2-fold: (1) to map the existing research landscape related to the soil salinization and plant-soil interactions, and (2) to highlight the significance of these interactions in mitigating the adverse effects of soil salinization under climate change. Through this analysis, we aim to seed future research directions that not only address the gaps identified but also contribute to the development of integrated and sustainable solutions for soil salinization challenges exacerbated by climate change.

## Methodology

### Description of the bibliometric analysis method using VOSviewer

A comprehensive bibliometric analysis of the scientific literature pertaining to soil salinization and plant-soil interactions was conducted utilizing VOSviewer (version 1.6.20), a robust instrument designed for the visualization and examination of trends and patterns within academic research.^17^ This tool aids in identifying principal nodes of research activity, uncovering emerging trends, and delineating potential avenues for future investigation through the generation of network maps of cited references, co-citation clusters, and keyword co-occurrence networks. The methodology adheres to the principles of scientometrics and capitalizes on the functionalities of VOSviewer to navigate the intricate terrain of interdisciplinary research encompassing soil science, plant biology, and climate change studies.

### Criteria for selecting databases and search terms

Literature search was conducted in the Web of Science in April 2024 using the following search terms: (TS = “salinization” or TS = “soil salinity”) and ([TS = “plant” or TS = “vegetation”] and [TS = “bacteria” or TS = “mycorrhiza” or TS = “fungi” or TS = “soil microbe”]) and (TS = “climate change” or TS = “warming” or TS = “drought” or TS = “precipitation” or TS = “moisture”). The literature covers the period from 1986 to 2024, totally have 169 papers. However, since there were only 11 papers published before 2013 (5 in 2013, 1 in 2012, 2 in 2009, 1 in 2008, 1 in 1996, and 1 in 1986), we used 2013 as a cutoff point to present the annual publication data from 2013 onward.

### Analytical techniques to identify trends, gaps, and clusters

The analytical approach entailed a multi-step process to meticulously examine the collected literature. The initial phase comprised a temporal analysis aimed at delineating the evolution of research volume over time. This was followed by a keyword co-occurrence analysis to identify the most prevalently discussed concepts, thereby unveiling dominant themes and discerning emerging trends within the field.^18^ Moreover, cluster analysis was employed to delineate distinct research areas and elucidate their interconnections.^19^ This comprehensive suite of analytical tools facilitated the mapping of the intellectual landscape, revealing research trends, gaps, and clusters within the global discourse on soil salinization and plant-soil interactions amid the changing climate.

## Part I: Bibliometric analysis

### Publication numbers and distribution

**Figure 2:**
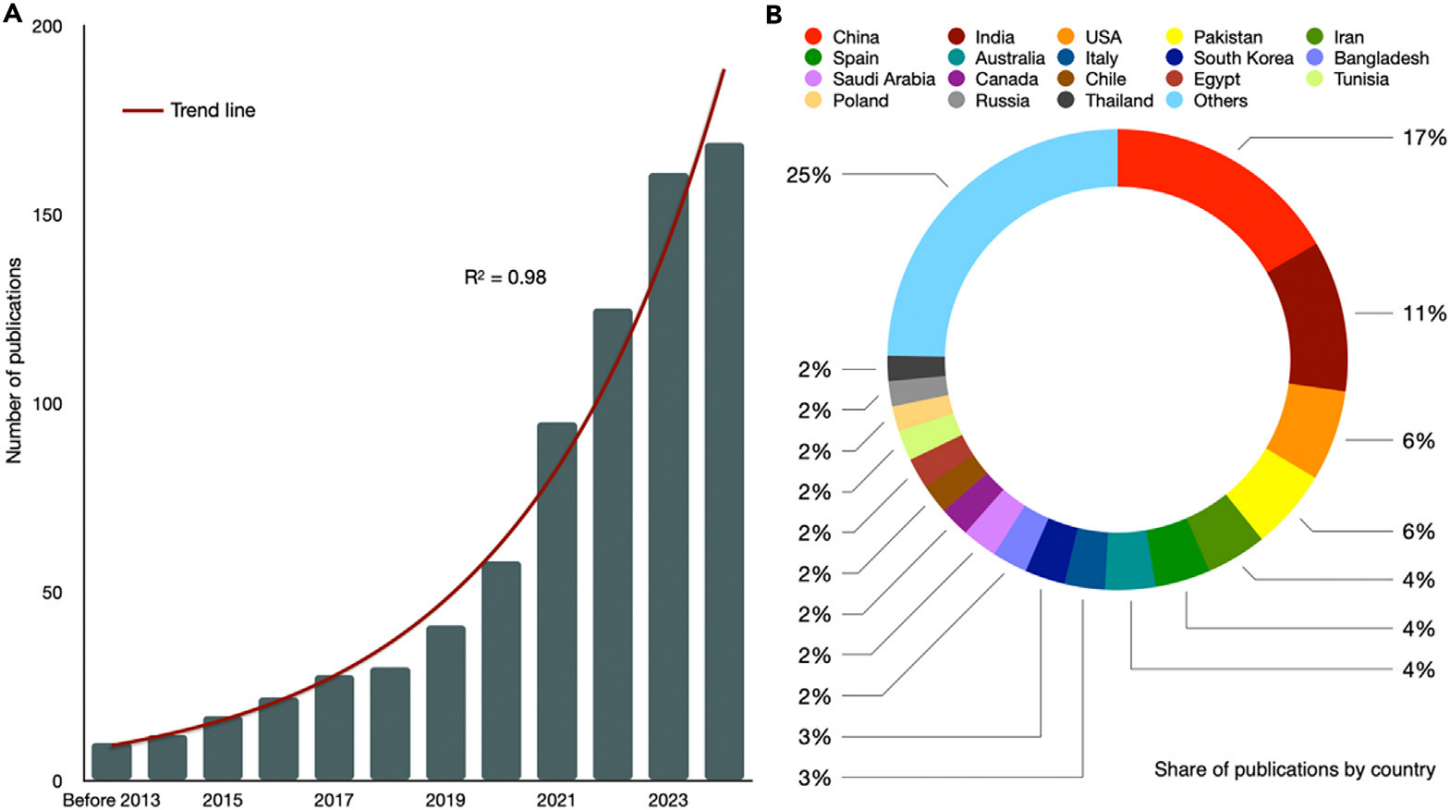
Global trends in publications (A) and distribution by country (B) on the mechanism of soil salinization The different color represents the major class from the cluster analysis.

Based on topic word searches, research on soil salinization and related plant-soil mechanisms shows a year-over-year upward trend. Exponential correlation modeling reveals a correlation coefficient (R^2^) of 0.98, indicating a strong relationship (Figure 2A). The results indicate that, prior to 2013, only 11 relevant papers were published, appearing in 1986, 1996, 2008, 2009, 2012, and 2013. From 2014 to 2024, there have been publications each year, totaling just 169 papers. This suggests that while research on plant-soil interactions in the context of soil salinization has garnered attention, it remains relatively limited. To further validate our findings, we conducted searches using individual topic keywords. We found 6,053 papers related to plant and soil salinization ([TS = “salinization” or TS = “soil salinity”] and [TS = “plant” or TS = “vegetation”]), 1,055 papers on microbes and soil salinization ([TS = “salinization” or TS = “soil salinity”] and [TS = “bacteria” or TS = “mycorrhiza” or TS = “fungi” or TS = “soil microbe”]), and 4,314 papers on climate change and soil salinization ([TS = “salinization” or TS = “soil salinity”] and [TS = “climate change” or TS = “warming” or TS = “drought” or TS = “precipitation” or TS = “moisture”]). These results further support our conclusion that research on the mechanisms of plant-soil interactions in soil salinization remains relatively underexplored.

Analysis of publication volumes by country reveals that China, India, and the United States lead in the number of published articles, accounting for one-third of the global total. This high publication output reflects the significant impact of soil salinization on agricultural productivity and food security in these countries, driving them to actively conduct research to address this issue. Additionally, countries such as Pakistan, Iran, South Africa, Bangladesh, and Saudi Arabia also contribute 2–6% of the total publications (Figure 2B). This indicates that, while major economies dominate soil salinization research, other countries facing the threat of salinization are also actively investing in related research. This further highlights the widespread impact of soil salinization on a global scale, particularly in countries heavily reliant on agriculture. Therefore, it is crucial to further promote global knowledge sharing and technological exchange to address this common environmental challenge.

### Co-occurrence and cluster analysis

**Figure 3:**
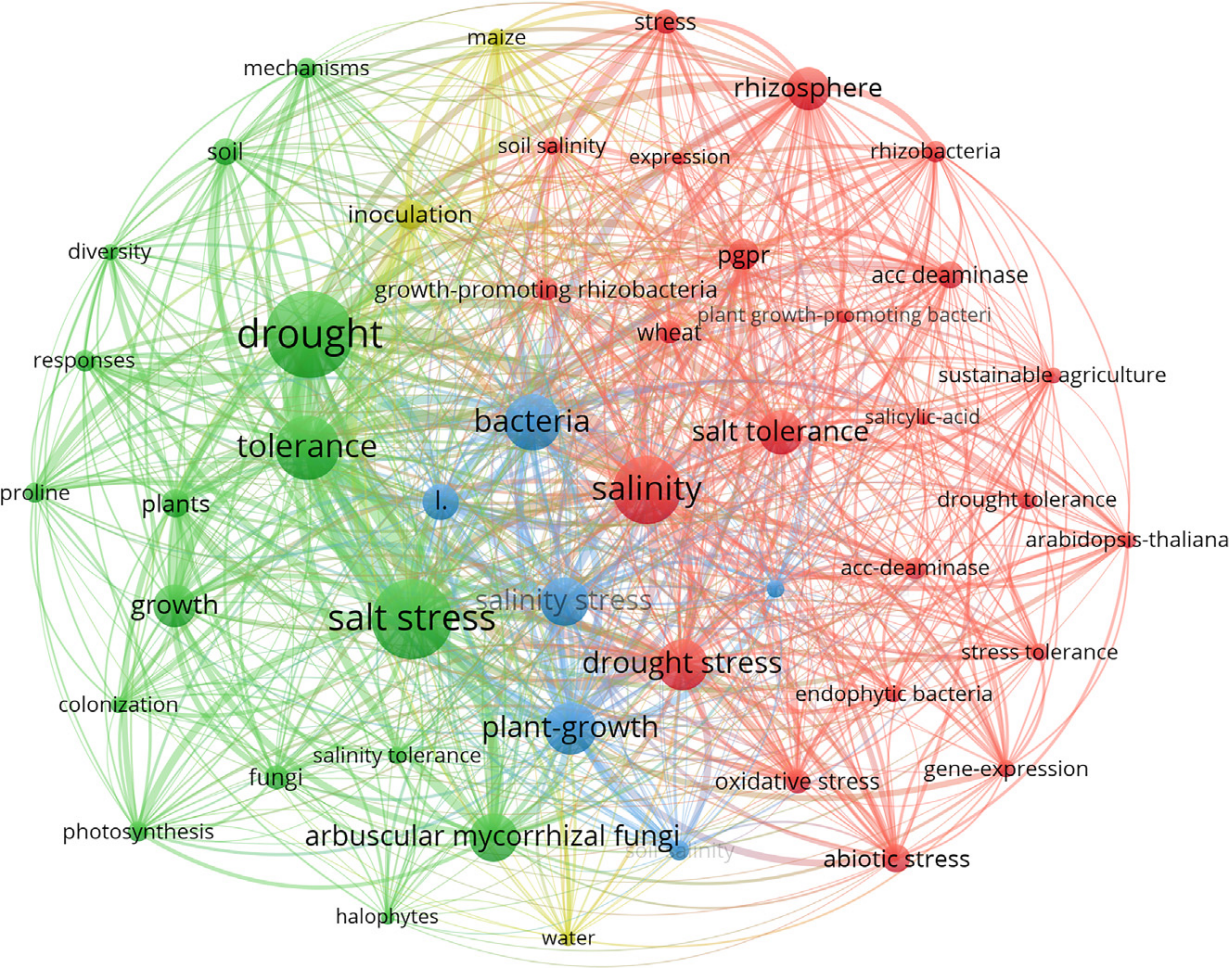
Network visualization of co-occurrence and cluster analysis regarding the mechanisms of soil salinization

The keyword co-occurrence analysis in soil salinization-related research highlights a primary focus on soil drought, salt stress, soil microbial community, and salt tolerance among other related areas of study. Furthermore, through cluster analysis, the keywords can be categorized into four major classes: plant growth mechanisms, soil microbiology, plant salt stress tolerance mechanisms (Figure 3). This categorization highlights the interdisciplinary nature of soil salinization research, encompassing both biological processes and practical approaches to mitigating salinity’s adverse effects. Interestingly, the analysis shows that studies focusing on soil-microbe interactions are relatively less frequent compared to other areas. This gap suggests that while the role of plants and their physiological responses to salinity stress has been extensively studied, the equally crucial role of soil microbial communities in mediating these interactions has not received as much attention. Soil microbes play a vital role in nutrient cycling, soil structure, and overall soil health, all of which are critical in saline environments.

The emergence of the keyword “Inoculation” in the context of plant-soil interactions is particularly noteworthy (Figure 3). Inoculation refers to the introduction of beneficial microbes, such as bacteria or fungi, into the plant rhizosphere to enhance plant tolerance to salinity stress. This strategy has gained increasing attention as a sustainable approach to mitigating soil salinization. Beneficial microbes, including plant growth-promoting rhizobacteria (PGPR) and mycorrhizal fungi, have been shown to significantly improve plant water uptake, nutrient assimilation, and stress resilience. By enhancing these processes, inoculation can alleviate the negative impacts of soil salinization on plant growth, leading to more resilient agricultural systems. This finding underscores the potential of harnessing microbial allies in the fight against soil salinization. However, it also points to the need for further research in this area, particularly to understand the specific mechanisms by which these microbes confer stress tolerance and how they interact with different plant species under varying environmental conditions. Expanding research on soil-microbe interactions could lead to more targeted and effective inoculation strategies, thereby enhancing the overall resilience of agricultural systems in saline-prone areas.

## Part II: A conceptual framework for climate change-induced soil salinization and plant-soil tolerance mechanisms

### Climate change-induced soil salinization

Climate change significantly impacts soil salinity dynamics through alterations in hydrological and temperature regimes.^2^^,^^8^ Key hydrological changes, such as variations in precipitation patterns including rainfall amount, frequency, intensity, and distribution, directly affect soil moisture, and salinity levels.^2^ These changes influence the processes of leaching—the removal of salts from the soil via water percolation or drainage—and evaporation, the loss of water from the soil through vaporization or transpiration.^20^ While higher leaching rates can decrease soil salinity by removing salts, increased evaporation rates tend to concentrate salts in the soil, thereby elevating soil salinity.

Erratic or reduced precipitation can exacerbate soil salinization by promoting evaporation and salt accumulation, especially when water inputs from rainfall do not suffice to counterbalance water losses through evaporation or transpiration, resulting in a net increase in soil salinity.^21^ Conversely, increased or intense precipitation may either dilute or mobilize salts depending on whether rainfall inputs exceed evaporation losses, potentially reducing soil salinity through dilution and leaching, or conversely, increasing it by mobilizing salts from deeper layers or external sources into the root zone.^21^^,^^22^

Temperature changes, induced by climate change, further complicate soil salinity dynamics by influencing evaporation rates and microbial activities in the soil.^23^ Rising temperatures can elevate evaporation rates, leading to increased soil salinity due to higher water loss relative to inputs.^5^^,^^24^ This process can result in the formation of salt crusts on the soil surface, negatively impacting soil physical properties and plant growth.^25^ Additionally, increased temperatures boost microbial metabolic rates and enzyme activities, potentially enhancing the decomposition of organic matter, nutrient cycling, and carbon sequestration.^26^ While these microbial processes can mitigate soil salinization by transforming salts into less soluble forms, they may also intensify salinization under conditions of elevated soil salinity and water stress, thereby inhibiting microbial activity and exacerbating salt accumulation.^27^

### Plant-soil tolerance mechanisms

#### Salt exclusion and tolerance mechanisms in plants

**Figure 4:**
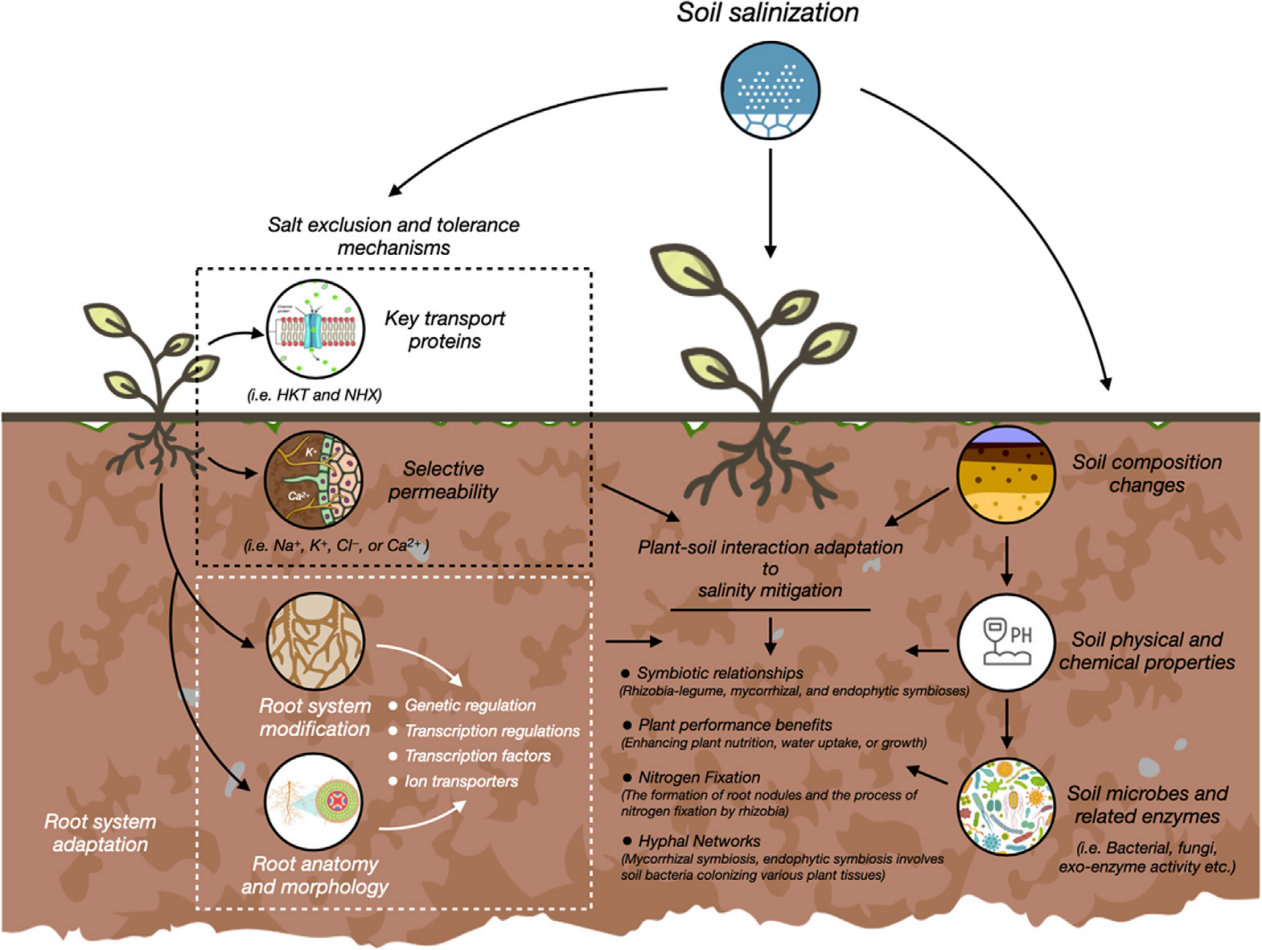
Plant-soil interaction mechanisms for salinity mitigation This figure highlights the diverse mechanisms plants employ to manage salt stress through intricate interactions with the soil. These mechanisms operate at multiple levels: cellular and tissue, organ and whole-plant, and community and ecosystem. At the cellular level, salt exclusion strategies regulate the movement of ions, with key roles played by proteins like HKT and NHX. On the organ and whole-plant scale, root system adaptations, guided by proteins such as ARF and SOS1, influence salt tolerance by modifying root traits. In the broader community and ecosystem context, plants engage in symbiotic relationships with soil microorganisms, such as rhizobia-legume, mycorrhizal, and endophytic symbioses, which enhance nutrition, water uptake, and growth.

Plants use to cope with salt stress at the cellular and tissue levels is salt exclusion, which prevents or reduces the entry of salt into the plant from the soil solution (Figure 4). Salt exclusion is mainly based on the selective permeability of the plasma membrane or the cell wall to different ions, such as Na^+^, K^+^, Cl^−^, or Ca^2+^.^28^^,^^29^ Various proteins that act as transporters, channels, pumps, or enzymes regulate the movement of ions across these barriers under saline conditions.^30^ For example, the high-affinity potassium transporter (HKT) is responsible for Na^+^ uptake and transport in roots. Depending on its subfamily and isoform, HKT can function as a Na^+^/K^+^ symporter or a Na^+^ uniporter. HKT can mediate salt exclusion by removing Na^+^ from the xylem sap and returning it to the soil solution or sequestering it into the vacuole.^31^^,^^32^ In contrast, the sodium/hydrogen exchanger (NHX) is responsible for Na^+^/H^+^ exchange across membranes.^33^ Depending on its subfamily and isoform, NHX can function as a Na^+^/H^+^ antiporter or a K^+^/H^+^ antiporter. NHX can mediate salt exclusion by extruding Na^+^ from the cytosol to the apoplast or sequestering it into the vacuole.^34^^,^^35^ Both HKT and NHX are regulated by various signaling pathways and transcription factors that modulate their expression and activity in response to salt stress (Figure 4). Different plant species express different isoforms of these proteins that confer different levels of salt tolerance.^36^ For instance, a salt-tolerant rice cultivar (Nona Bokra) expresses a HKT1; 5 isoform that reduces Na^+^ accumulation in the shoot, while a salt-sensitive rice cultivar (Koshihikari) expresses a different HKT1; 5 isoform that increases Na^+^ accumulation in the shoot.^31^^,^^37^.

#### Root system adaptations for salt dilution and excretion

Root system adaptation aims to increase the water uptake or decrease the salt uptake by roots from the soil solution by developing deeper or more extensive root systems that can access fresh water sources or avoid saline zones in soils, or modifying root anatomy or morphology to facilitate salt excretion through specialized structures such as salt glands or bladders.^38^^,^^39^ These adaptations are regulated by various genes, hormones, or signals that modulate root development, growth, or differentiation under salt stress.^40^ For instance, the auxin response factor (ARF) is a transcription factor that regulates auxin-responsive gene expression and root morphology.^41^^,^^42^ Depending on its domain structure and interaction partners, ARF can function as an activator or a repressor of auxin-responsive genes. ARF can mediate root system adaptation by modulating root length, depth, density, diameter, branching, or architecture under salt stress.^43^ In contrast, the sodium transporter (SOS1) is a plasma membrane-localized cation transporter that regulates Na^+^ efflux from roots.^44^^,^^45^ Depending on its subfamily and isoform, SOS1 can function as a Na^+^/H^+^ antiporter or a Na^+^/K^+^ symporter. SOS1 can mediate root system adaptation by facilitating salt excretion through specialized structures such as salt glands or bladders under salt stress.^46^ Both ARF and SOS1 are influenced by various signaling pathways and transcription factors that modulate their expression and activity in response to salt stress.^47^ Different plant species express different isoforms of these proteins that confer different levels of salt tolerance. For example, a salt-tolerant tomato cultivar (LA2714) expresses a ARF7 isoform that increases root length and density under salt stress, while a salt-sensitive tomato cultivar (Moneymaker) expresses a different ARF7 isoform that decreases root length and density under salt stress.^34^^,^^35^^,^^48^^,^^49^.

#### The role of plant-microbe interactions in salinity management

Plants cope with salt stress at the community and ecosystem levels is by forming symbiotic relationships with soil microorganisms that benefit both parties in exchange for resources or signals^50^ (Figure 4). These relationships include rhizobia-legume, mycorrhizal, endophytic, or cyanobacterial symbioses, which can improve plant performance under salt stress by enhancing plant nutrition, water uptake, growth, or quality.^51^^,^^52^ For example, rhizobia-legume symbiosis is the association between nitrogen-fixing bacteria (rhizobia) and leguminous plants (such as beans, peas, or clovers) that form root nodules where nitrogen fixation occurs.^53^ This association can provide nitrogen to plants under nitrogen-deficient conditions, which is a vital nutrient for plant growth and development but often scarce or unavailable in saline soils.^54^^,^^55^ In contrast, mycorrhizal symbiosis is the association between soil fungi (mycorrhizae) and most land plants (such as cereals, vegetables, or fruits) that form hyphal networks that connect plant roots with soil particles.^56^ This symbiosis can improve water uptake efficiency under water-deficient conditions, which is a crucial resource for plant growth and development but often scarce or unavailable in saline soils.^23^^,^^57^ Furthermore, endophytic symbiosis is the association between soil bacteria (endophytes) and most land plants (such as grasses, cereals, or trees) that colonize the internal tissues of roots, stems, leaves, or seeds without causing any apparent harm to the host.^58^ This association can produce plant growth regulators, antioxidants, osmolytes, or enzymes that modulate plant physiology or metabolism under salt stress.^59^

## Discussion on research gaps: How plant-soil interactions mitigate the climate change-induced soil salinization

### Comprehensive analysis of plant-soil synergies under climate-induced salinization

The nexus between plant and soil systems, particularly under the exacerbating influence of climate-induced soil salinization, emerges as a critical yet underexplored domain within environmental research.^5^ Although initial inquiries have illuminated the capacity of plant-soil interactions to counter salinization, the intricacies of the regulatory mechanisms operating under the duress of climate change necessitate further elucidation.^21^^,^^23^ To illustrate this, we can consider specific studies where plant-soil interactions have been shown to modulate salinity tolerance. For instance, research on Arabidopsis thaliana has demonstrated how certain soil microbes can induce the expression of stress-responsive genes that enhance the plant’s ability to cope with saline conditions.^60^ These findings suggest that understanding the genetic and molecular mechanisms at play is essential for developing comprehensive strategies to combat soil salinization. This gap underscores an imperative need for an integrated exploration into the nuanced modulation of plant responses to salinity by these interactions and their contribution to enhancing soil resilience against salinization.^61^ The complexity inherent in these mechanisms encompasses a broad spectrum of biological, chemical, and physical processes, thereby presenting an expansive challenge for current scientific investigations.

A pivotal aspect of advancing comprehension within this field lies in recognizing the interconnected impacts of climate change on soil salinization, which are intricately linked with dynamic responses from plant-soil systems.^62^ These responses are orchestrated through a series of mechanisms, including the selective permeability of plant cellular membranes to specific ions, the pivotal role of soil microbiota in augmenting plant salinity tolerance, and the evolutionary adaptations of root systems designed to minimize salt assimilation.^63^ For example, studies have shown that soil microbes like Pseudomonas species can enhance ion transport in plants under saline conditions, thereby improving salt tolerance.^64^ Such examples underscore the need for a more detailed exploration of how these microbial interactions can be leveraged to develop new agricultural practices. Investigating the modulation of these processes in the face of escalated soil salinity due to climate change demands a meticulous and detailed approach.

Moreover, the scarcity of longitudinal studies examining the persistent impacts of plant-soil interactions in mitigating soil salinization accentuates a significant research trajectory.^65^ Future endeavors must transcend the immediate physiological responses of plants to salinity stress to investigate the enduring implications of these interactions. This encompasses evaluating the adaptability of plant-soil systems to fluctuating climatic conditions and discerning the utility of these adaptations in formulating sustainable soil management strategies.

Addressing these multifaceted complexities mandates the adoption of an interdisciplinary methodology that integrates knowledge from soil science, plant physiology, microbiology, and climate science. Such a comprehensive approach is quintessential for devising predictive models capable of assessing the repercussions of plant-soil interactions across diverse climatic scenarios. It is through these rigorous, cohesive research efforts that the potential of plant-soil synergies can be fully realized, offering sustainable resolutions to the burgeoning challenges of soil salinization in the climate change epoch.

### Deep-dive into mechanistic insights through advanced techniques

Advancements in genomic and transcriptomic technologies have revolutionized the scientific community’s ability to decode the molecular intricacies of how plants and their rhizosphere microbiota respond to salinity stress.^66^^,^^67^ These technologies allow for an unprecedented exploration of the genetic and molecular fabric of salinity tolerance, revealing the orchestration of gene expression, protein function, and metabolic pathways in response to saline environments.^68^ For example, transcriptomic studies on rice (*Oryza sativa*) have identified specific genes responsible for the synthesis of osmoprotectants, which play a crucial role in osmotic stress management.^69^ Similarly, genomic studies on barley (*Hordeum vulgare*) have pinpointed genetic variants associated with ion transporters that are critical for salinity tolerance.^70^ Furthermore, transcriptomic profiling can pinpoint specific stress-responsive genes that are upregulated in plants to manage osmotic stress, such as those encoding for osmoprotectants or ion transporters.^71^^,^^72^ Genomic analyses extend these findings by mapping the genetic variants that contribute to phenotypic traits associated with salinity tolerance, offering potential markers for breeding programs.

The “inoculation” of plants with salt-tolerant microbes is emerging as a transformative approach to bolster plant resilience against salinity. This technique leverages the symbiotic relationships between plants and beneficial microbes, which can enhance nutrient uptake, improve water efficiency, and induce systemic resistance to stress.^73^ Research is increasingly focusing on the identification of microbial strains that possess specific mechanisms for supporting plant health in saline conditions, such as the production of phytohormones or the solubilization of phosphorus, which can significantly alleviate the physiological burden of salt stress on plants.^74^^,^^75^ To further support this discussion, we have included a summary table that outlines key studies on “inoculation” techniques, listing the plant species involved, the specific microbes used, and the observed beneficial effects (Table 1).

**Table 1:** Summary of key studies on “inoculation” techniques in soil salinization

Plant species	Microbe used	Technique employed	Observed beneficial effect	Reference
Rice (*Oryza sativa*)	*Pseudomonas fluorescens*	Bacterial Inoculation	Enhanced salt tolerance through improved ion transport, increased root growth	Gupta et al., (2023)^69^;
Wheat (*Triticum aestivum*)	*Bacillus subtilis*	Rhizobacteria Inoculation	Increased phosphorus solubilization, higher grain yield, enhanced drought resistance	Zahra et al., (2023)^76^
Barley (*Hordeum vulgare*)	*Azospirillum brasilense*	Inoculation with PGPR	Improved osmotic balance, increased proline accumulation, enhanced stress resilience	Chieb & Gachomo (2023)^70^
Tomato (*Solanum lycopersicum*)	*Glomus intraradices* (Mycorrhiza)	Fungal Inoculation	Enhanced nutrient uptake, increased water use efficiency, improved plant growth under drought	Zhang et al., (2024)^77^
Maize (*Zea mays*)	*Rhizobium* spp.	Bacterial-Fungal Co-Inoculation	Synergistic effects on plant growth, reduced Na^+^ accumulation, improved chlorophyll content	Brambilla et al., (2022)^78^
Sunflower (Helianthus annuus)	*Bacillus licheniformis*	Bacterial Inoculation	Increased plant biomass, improved root architecture, enhanced nutrient uptake	Khoso et al., (2024)^79^
Soybean (Glycine max)	*Bradyrhizobium japonicum*	Rhizobacteria Inoculation	Improved nitrogen fixation, higher seed protein content, enhanced salinity tolerance	Riviezzi et al., (2020)^80^

Note: PGPR-plant growth-promoting rhizobacteria.

In the context of plant-soil-microbial systems, the application of advanced research techniques is shedding light on the complex, bidirectional interactions that govern the resilience of these systems to salinity. For instance, understanding how plants can selectively enhance the recruitment of beneficial microbes under saline conditions, or how microbes can modulate plant gene expression to enhance salt tolerance, are areas ripe for exploration.^81^^,^^82^^,^^83^ Such insights are critical for designing integrated management strategies that harness the full potential of plant and microbial adaptations to combat soil salinization.

By pushing the boundaries of the mechanistic understanding through the integration of genomic, transcriptomic, and microbiological research, current studies are laying the foundation for innovative solutions to soil salinization. These solutions, grounded in a deep understanding of the underlying biological processes, have the potential to transform agricultural practices and ecosystem management strategies, making them more resilient to the challenges posed by an increasingly saline world. This concerted effort toward unraveling and leveraging the natural resilience mechanisms within plant-soil-microbial systems represents a critical step toward sustainable salinity management and the preservation of agricultural and natural ecosystems in the face of escalating environmental stressors.

## Implications

This review highlights the growing challenge of soil salinization, which is increasingly driven by climate change. It emphasizes the crucial role of plant-soil interactions in developing resilience and strategies for mitigating salinity stress. Advances in genomic and transcriptomic research have provided valuable insights into the molecular mechanisms by which plants and microbes respond to salinity. These findings offer promising directions for breeding salt-tolerant crops and developing microbial inoculants. By understanding the physiological and biochemical strategies that plants and their associated microbiomes employ, there is significant potential to improve soil salinity management practices. However, there are still important research gaps, particularly regarding the long-term effects of plant-soil interactions on soil salinity under different climate scenarios. The challenge also lies in translating these molecular insights into practical agricultural applications. Addressing these issues requires a multidisciplinary approach that brings together expertise in soil science, plant physiology, microbiology, and climate change research. Future work should focus on developing predictive models that simulate plant-soil interactions under salinity stress, which will be essential for planning effective mitigation and adaptation strategies in the face of climate change.

## Conclusion

Our review calls for a coordinated global research effort to better understand and utilize plant, soil, and microbial adaptations in addressing soil salinization. By focusing on sustainable and innovative solutions, such efforts can contribute to enhancing food security, strengthening ecosystem resilience, and mitigating the effects of climate change. Additionally, there is a pressing need for research that bridges the gap between molecular discoveries and practical applications, ensuring that agricultural systems remain sustainable and that ecosystem functions are preserved in saline environments.

## Acknowledgments

This study was funded by the Major Science and Technology Innovation Pilot Project for Water Resources Protection and Integrated-Saving Utilization in the Yellow River Basin of Inner Mongolia Autonomous Region (2023JBGS0007), Natural Science Foundation of Inner Mongolia A. R. of China (2023QN04001), Start-up Funding from 10.13039/501100003850Inner Mongolia University (21800-5223728), the Science and Technology Research Program of 10.13039/501100007957Chongqing Municipal Education Commission (KJQN202300541), Start-up funding from 10.13039/100010338Chongqing Normal University (202305000221), and Start-up funding from 10.13039/501100016349Sichuan Normal University (XJ20210431).

## Author contributions

H.T.: conceptualization, methodology, resources, visualization, writing—original draft, writing—review and editing; L.D.: conceptualization, resources, and visualization; C.X.: visualization, funding acquisition, writing—review and editing, supervision; J.L.: conceptualization, methodology, writing—original draft, writing—review and editing, supervision, funding acquisition.

## Declaration of interests

The authors declare no competing interests.
